# Peripheral Blood Eosinophil as a Predictor of Clinical Outcomes in Acute Exacerbation of Chronic Obstructive Pulmonary Disease: A Cross-Sectional Study From a Tertiary Care Center in South India

**DOI:** 10.7759/cureus.90638

**Published:** 2025-08-21

**Authors:** Pedada Mounika, Elen Ann Abraham, Ghanshyam Verma, AkhilAnand PG, Keerthana Prakash, Ragavi Elango, Aldrin Santhosh, Pavithra C

**Affiliations:** 1 Department of Respiratory Medicine, Sree Balaji Medical College and Hospital, Chennai, IND

**Keywords:** aecopd, blood eosinophils, chronic obstructive pulmonary disease, clinical outcomes, copd exacerbation, corticosteroid response

## Abstract

Aim and objective

To evaluate the relationship between peripheral blood eosinophil levels (>5% at admission) and clinical outcomes, including ICU admission, duration of hospitalization, need for mechanical ventilation, and corticosteroid responsiveness, in patients hospitalized with acute exacerbation of chronic obstructive pulmonary disease (AECOPD) at Sree Balaji Medical College and Hospital, Chennai.

Methods

A total of 120 patients aged >45 years with AECOPD were enrolled in this study. Peripheral blood eosinophil percentage at the time of admission was recorded for all participants. Based on eosinophil levels, patients were categorized into two groups: eosinophilic (>5%) and non-eosinophilic (≤5%). Clinical outcomes, including ICU admission, duration of hospital stay, requirement for mechanical ventilation, and corticosteroid use, were analyzed. Statistical analysis was performed using the chi-square test and regression analysis to determine associations between eosinophil levels and clinical parameters.

Results

Patients in the non-eosinophilic group demonstrated significantly higher rates of ICU admissions (21.7% vs. 9.4%, p = 0.018), longer hospital stays (7.9 ± 2.4 vs. 5.6 ± 1.9 days, p = 0.003), and greater need for invasive ventilation (13.3% vs. 4.6%, p = 0.024). Corticosteroid usage was also notably higher in the non-eosinophilic group (73.3% vs. 42.1%, p < 0.001). Although mortality was slightly elevated in the non-eosinophilic group (5.7% vs. 2.1%), the difference was not statistically significant (p = 0.28).

Conclusion

Peripheral blood eosinophil count >5% is associated with favorable clinical outcomes in patients hospitalized with AECOPD. As a cost-effective and readily available biomarker, eosinophil levels may aid in stratifying patients and guiding personalized corticosteroid therapy during exacerbations.

## Introduction

Chronic obstructive pulmonary disease (COPD) is a leading cause of respiratory morbidity and mortality worldwide. Acute exacerbations of COPD (AECOPD) substantially influence disease progression by increasing hospitalizations, healthcare burden, and mortality risk [1]. Identifying reliable biomarkers that can stratify patients and predict outcomes during exacerbations remains a key clinical priority [2].

Eosinophilic inflammation is a well-recognized phenotype in stable COPD and has been associated with a favorable response to corticosteroid therapy [3]. Peripheral blood eosinophil count, which serves as a surrogate marker for airway eosinophilia, is inexpensive and routinely available. However, its predictive utility during acute exacerbations remains debated, with studies reporting conflicting results [4]. Evidence suggests that eosinophilic AECOPD may be associated with a milder clinical course, lower risk of bacterial infection, and better corticosteroid responsiveness, whereas non-eosinophilic exacerbations are often linked to infections and worse prognosis [5]. The cutoff value for defining eosinophilia has varied across studies, ranging from 2% to 5%. While some studies support the lower threshold for sensitivity, others demonstrate stronger prognostic value at higher cutoffs [6,7].

In this context, we adopted a cutoff of >5% peripheral blood eosinophils at admission, which has been used in prior studies to discriminate eosinophilic from non-eosinophilic exacerbations and to assess their impact on outcomes. This threshold may also be more practical in resource-limited settings, where clinical decisions need to be guided by easily interpretable laboratory markers. This study aimed to assess whether a peripheral blood eosinophil count >5% at admission predicts clinical outcomes, including ICU admission, length of hospital stay, need for mechanical ventilation, and corticosteroid responsiveness, in patients hospitalized with AECOPD. The findings are expected to provide insights into the role of eosinophils in prognosticating exacerbation severity and guiding treatment strategies.

## Materials and methods

Study design and setting

This cross-sectional, observational study was conducted in the Department of Respiratory Medicine, Sree Balaji Medical College and Hospital, Chennai, between January 2023 and December 2024. The study aimed to evaluate the relationship between peripheral blood eosinophil levels and clinical outcomes in patients admitted with AECOPD. As this was a retrospective analysis of anonymized hospital records, no additional patient contact or intervention was required. Written informed consent for the use of anonymized clinical data in research had been obtained from all patients at the time of admission, in accordance with institutional policy. The study was granted exemption by the Institutional Ethics Committee, Sree Balaji Medical College and Hospital (Exemption No.: IEC/EXEMPTION/2025/034).

Study population

A total of 120 patients aged >45 years admitted with a diagnosis of AECOPD during the study period were included. COPD diagnosis was confirmed by spirometry (post-bronchodilator forced expiratory volume in one second (FEV₁)/forced vital capacity (FVC) <70% and bronchodilator reversibility <12%). AECOPD was defined as an acute worsening of respiratory symptoms (increased dyspnea, cough, and/or sputum volume/purulence) requiring systemic therapy. Patients were recruited using consecutive sampling. Exclusion criteria included co-existing asthma, bronchiectasis, pneumonia, pulmonary tuberculosis, malignancy, autoimmune or hematological disorders, or incomplete hospital records.

Grouping based on eosinophil count

Peripheral blood eosinophil percentage was obtained from a complete blood count (CBC) performed at the time of hospital admission. Counts were measured using an automated hematology analyzer (Sysmex XN-1000, Kobe, Japan), which was subjected to daily calibration and internal quality control. Patients were grouped as follows: eosinophilic AECOPD (eosinophil percentage >5%) and non-eosinophilic AECOPD (eosinophil percentage ≤5%).

Clinical outcomes

The following outcomes were analyzed: ICU admission - requirement for transfer to ICU due to respiratory failure or hemodynamic instability; hospital stay duration - number of days from admission to discharge; need for invasive mechanical ventilation; systemic corticosteroid use - standardized protocol of IV methylprednisolone 40 mg/day with tapering based on clinical response; in-hospital mortality.

Patients with missing outcome data were excluded to ensure data completeness.

Statistical analysis

Data were analyzed using SPSS version 26.0 (IBM Corp., Armonk, NY). Continuous variables were expressed as mean ± standard deviation (SD) and compared using Student’s t-test. Categorical variables were summarized as frequencies and percentages, with comparisons performed using the chi-square test or Fisher’s exact test as appropriate. A p-value <0.05 was considered statistically significant. No multivariate regression analysis was performed, and the text has been corrected accordingly to ensure consistency.

## Results

A total of 120 patients admitted with AECOPD were included, of whom 47 (39.2%) were classified as eosinophilic (peripheral eosinophil percentage >5%) and 73 (60.8%) as non-eosinophilic (≤5%).

Clinical outcomes

Patients in the eosinophilic group demonstrated significantly more favorable outcomes than those in the non-eosinophilic group.

ICU admission occurred in 9.4% (n = 4) of eosinophilic patients compared to 21.7% (n = 16) in the non-eosinophilic group (χ² = 5.57, p = 0.018; risk difference = -12.3%, 95% CI: -22.8 to -1.9).

Hospital stay was significantly shorter in the eosinophilic group (5.6 ± 1.9 days) compared to the non-eosinophilic group (7.9 ± 2.4 days; t = -5.29, p = 0.003; mean difference = -2.3 days, 95% CI: -3.8 to -0.9).

Invasive ventilation was required in 4.6% (n = 2) of eosinophilic patients versus 13.3% (n = 10) of non-eosinophilic patients (χ² = 5.09, p = 0.024; risk difference = -8.7%, 95% CI: -16.4 to -1.0).

Systemic corticosteroid use was significantly lower in eosinophilic patients (42.1%, n = 20) compared to non-eosinophilic patients (73.3%, n = 54; χ² = 13.98, p < 0.001; risk difference = -31.2%, 95% CI: -46.5 to -15.9).

In-hospital mortality was observed in 2.1% (n = 1) of eosinophilic patients and 5.7% (n = 4) of non-eosinophilic patients, but this difference was not statistically significant (χ² = 1.15, p = 0.28) (Table 1).

**Table 1:** Comparison of clinical outcomes between eosinophilic and non-eosinophilic groups (n = 120). Continuous variables are expressed as mean ± SD and compared using Student’s t-test. Categorical variables are expressed as % (n) and compared using the chi-square test. * A p < 0.05 was considered statistically significant. RD = risk difference; MD = mean difference; CI = confidence interval; NS = not significant.

Parameter	Eosinophilic (n = 47)	Non-eosinophilic (n = 73)	Test statistic	p-value	95% CI/effect size
ICU admission	9.4% (n = 4)	21.7% (n = 16)	χ² = 5.57	0.018*	RD: –12.3% (–22.8 to –1.9)
Hospital stay (days)	5.6 ± 1.9	7.9 ± 2.4	t = –5.29	0.003*	MD: –2.3 (–3.8 to –0.9)
Invasive ventilation	4.6% (n = 2)	13.3% (n = 10)	χ² = 5.09	0.024*	RD: –8.7% (–16.4 to –1.0)
Steroid use	42.1% (n = 20)	73.3% (n = 54)	χ² = 13.98	<0.001*	RD: –31.2% (–46.5 to –15.9)
Mortality	2.1% (n = 1)	5.7% (n = 4)	χ² = 1.15	0.28	NS

These findings suggest that eosinophilic AECOPD is associated with less severe clinical courses in terms of ICU requirement, ventilation need, and hospitalization burden, although mortality outcomes did not differ significantly between groups.

**Figure 1:**
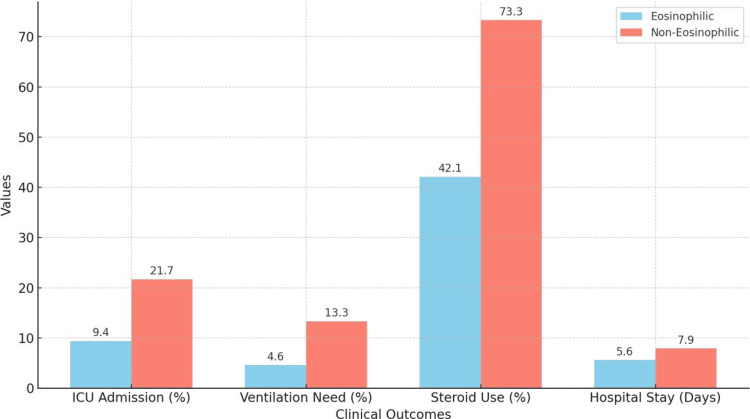
Comparison of clinical outcomes in eosinophilic and non-eosinophilic AECOPD patients. Bar chart comparing ICU admission (%), need for invasive ventilation (%), systemic corticosteroid use (%), and mean hospital stay (days) between eosinophilic (n = 47) and non-eosinophilic (n = 73) groups. Eosinophilic patients had significantly lower ICU admission, ventilation, and steroid use, and shorter hospital stays (p < 0.05 for all except mortality). AECOPD = acute exacerbations of chronic obstructive pulmonary disease.

The bar graph in Figure 1 illustrates the differences in key clinical outcomes between eosinophilic and non-eosinophilic groups among patients with AECOPD. The parameters compared include ICU admission rates, ventilation need, steroid use, and average hospital stay duration. The eosinophilic group demonstrated significantly lower rates of ICU admission (9.4% vs. 21.7%), ventilation need (4.6% vs. 13.3%), and steroid use (42.1% vs. 73.3%) compared to the non-eosinophilic group. The average hospital stay was also shorter in the eosinophilic group (5.6 ± 1.9 days vs. 7.9 ± 2.4 days). These findings suggest a more favorable clinical course in eosinophilic AECOPD patients.

## Discussion

This cross-sectional study evaluated the association between peripheral blood eosinophil levels and clinical outcomes among patients admitted with AECOPD. Our findings demonstrate that patients with eosinophil counts >5% had significantly better clinical outcomes, including reduced ICU admission rates, shorter hospital stays, decreased need for invasive ventilation, and lower systemic corticosteroid usage. Mortality rates were higher in the non-eosinophilic group, but the difference did not reach statistical significance.

These results are consistent with prior research highlighting the existence of distinct inflammatory phenotypes in AECOPD. Eosinophilic AECOPD is increasingly recognized as a treatable trait with a more favorable prognosis and enhanced responsiveness to corticosteroid therapy. Bafadhel et al. [4] and Vedel-Krogh et al. [1] demonstrated that patients with eosinophilic inflammation respond more robustly to steroids and exhibit reduced bacterial colonization, contributing to milder exacerbations. Similarly, Pascoe et al. [6] reported that higher eosinophil counts predicted improved outcomes with inhaled corticosteroids, supporting the role of eosinophils as a biomarker of steroid responsiveness.

In our cohort, non-eosinophilic patients exhibited significantly higher rates of ICU admissions and mechanical ventilation. This observation aligns with findings from Barnes [5], who noted that non-eosinophilic exacerbations are predominantly driven by neutrophilic inflammation and bacterial infections, resulting in more severe disease and a blunted response to corticosteroids. These exacerbations may necessitate alternative therapies such as antibiotics and adjunctive anti-inflammatory agents.

The mean duration of hospital stay in the eosinophilic group (5.6 ± 1.9 days) was significantly shorter compared to the non-eosinophilic group (7.9 ± 2.4 days; p = 0.003). Pavord et al. [7], in their meta-analysis, similarly demonstrated that eosinophil-guided therapy reduces hospital length of stay and readmission rates. This finding is of considerable clinical significance, as reduced hospitalization directly translates to lower healthcare costs and improved resource allocation, particularly in low- and middle-income settings. Although mortality was higher in the non-eosinophilic group (5.7% vs. 2.1%), the difference was not statistically significant (p = 0.28). Negewo et al. [8] suggested that while eosinophil counts are predictive of exacerbation severity and steroid responsiveness, their role in predicting mortality requires larger, prospective datasets to draw firm conclusions.

Biologically, eosinophilic inflammation in COPD is mediated by type 2 immune responses and interleukin-5 signaling, pathways known to be sensitive to corticosteroid therapy. In contrast, neutrophilic AECOPD involves activation of different inflammatory pathways, often necessitating antibiotics and potentially adjunctive therapies beyond corticosteroids [3]. This distinction has spurred interest in novel biologic agents such as mepolizumab and benralizumab, which target eosinophilic inflammation. Zeiger et al. [9] have shown promising results with these biologics in reducing exacerbations in eosinophilic COPD patients, suggesting future avenues for therapy. Couillard et al. [10] further emphasized that absolute eosinophil counts may provide additional prognostic value over percentages alone, particularly in severe exacerbations where leukocyte dynamics are altered. While our study used eosinophil percentages, future studies should evaluate absolute counts alongside other biomarkers such as C-reactive protein (CRP) and procalcitonin for a more comprehensive assessment.

This study has several notable strengths. First, it addresses a clinically relevant question of whether peripheral blood eosinophils can predict outcomes in AECOPD, using a clear and practical threshold (>5%). The study design involved consecutive recruitment in a real-world tertiary care setting, which enhanced its applicability. Outcomes assessed, including ICU admission, hospital stay, need for invasive ventilation, and corticosteroid responsiveness, are objective, routinely recorded, and clinically meaningful. Additionally, the use of a standardized corticosteroid protocol adds to methodological consistency.

The study also acknowledges important limitations. Being cross-sectional and retrospective, causal inference cannot be established. The single-center nature and modest sample size may limit external validity and statistical power, particularly for mortality outcomes. Microbiological data were not collected, precluding differentiation between bacterial and non-infectious exacerbations. Only eosinophil percentages were used, which may be influenced by leukocyte shifts during infection; absolute eosinophil counts might provide additional value. Comorbidities were not fully stratified, which may have acted as unmeasured confounders. Long-term outcomes such as readmission rates and exacerbation frequency were not captured. Despite these limitations, this study contributes valuable data from an underrepresented population in South India. It highlights eosinophilic AECOPD as a distinct phenotype associated with more favorable short-term outcomes and provides a strong rationale for multicenter, prospective studies incorporating absolute eosinophil counts, microbiological confirmation, and broader biomarker panels.

## Conclusions

Our findings support the growing body of evidence that peripheral blood eosinophil counts may serve as a practical biomarker for phenotyping AECOPD and anticipating short-term clinical outcomes. While not definitive, eosinophil-guided management holds promise for optimizing corticosteroid use and tailoring therapy within the framework of precision medicine. Future multicenter, prospective studies incorporating absolute eosinophil counts and additional biomarkers are warranted to validate these observations and strengthen their application in routine COPD care.
